# Reducing Antibiotic Misuse through the Use of Point-of-Care Tests in Germany: A Survey of 1257 Medical Practices

**DOI:** 10.3390/healthcare11172466

**Published:** 2023-09-04

**Authors:** Tina Peiter, Monika Haering, Spasenija Bradic, Graça Coutinho, Karel Kostev

**Affiliations:** 1 Reckitt Benckiser Deutschland GmbH, 69115 Heidelberg, Germany; monika.haering@reckitt.com (M.H.);; 2 Reckitt Benckiser Healthcare Ltd., Slough SL1 4AQ, UK; 3 University Hospital, Philipps-University, 35043 Marburg, Germany

**Keywords:** swab tests, point-of-care tests, antibiotic, acute pharyngitis, general practice

## Abstract

Though more than 80% of acute pharyngitis (AP) cases have a viral etiology, it remains one of the most common causes for the unnecessary prescription of antibiotics (ABs). Half of patients receive antibiotics in general practice. Point-of-Care Tests (POCTs) distinguish between bacterial and viral pharyngitis. The objective of this study was to evaluate the use of POCTs using throat swabs to detect β-Streptococcus pyogenes Group A (strep A) infection among patients with sore throat/acute pharyngitis in primary care practices across Germany. A study was conducted in 1257 primary care practices. Two questionnaires were administered concerning frequency, POCT results and whether antibiotics were prescribed. Of the 1257 physicians, 60% used POCTs. Of these, 25% used a POCT before prescribing an antibiotic, 39% in cases of severe sore throat, 40% in cases of long-lasting pharyngitis and 25% in other cases. In total, 83% considered the adoption of POCTs in everyday practice to be important or very important for the diagnosis of strep A, 90% considered it important or very important for achieving a more sensible use of antibiotics and the prevention of bacterial resistance and 80% considered it important or very important for justifying to patients whether or not an antibiotic is needed. POCT results and information on AB prescriptions were available for 583 patients. Of these, 22.5% tested positive for strep A, and 21.8% were prescribed antibiotics. Our study shows that the use of swab tests in patients with sore throat in primary care practices results in high levels of physician satisfaction and can strongly reduce the misuse of antibiotics in clinical practice.

## 1. Introduction

Acute pharyngitis (AP), characterized by sore throat, is one of the most common disorders and reasons for consultations with primary care practice (PCP) physicians, otolaryngologists and pediatricians in Germany [1]. Though AP has a viral etiology in 70–80% of cases, it remains one of the most common causes for the unnecessary prescription of antibiotics [2].

Kern and Kostev reported that in Germany, 46% of adult patients and 20% of children and adolescents with acute pharyngitis received AB prescriptions. Moreover, in both adults and children, AP was a diagnosis associated with four-times-higher odds of receiving an AB prescription [3]. The main negative consequence of inappropriate antibiotic use is antimicrobial resistance, which has been considered a global public health challenge for many years and is the cause of severe infections and increased mortality. Unfortunately, such resistant bacteria have not only been detected in hospital patients but also in patients in primary care [4]. Patients who receive an AB prescription in primary care for a respiratory infection often develop bacterial resistance to this antibiotic drug. Finally, patients who receive a second-line AB drug have an increased risk for the development of resistance to further AB drugs [5].

As symptom presentations of viral and bacterial AP are similar, achieving a valid differentiation of these forms is challenging in clinical practice [6]. For example, tonsillar exudate is considered a symptom what occurs in bacterial rather than viral AP; however, this symptom does not enable one to clearly distinguish bacterial from viral infection [7].

Point-of-Care tests (POCTs) represent a novel means of rapidly detecting viral and bacterial respiratory tract infections to avoid inappropriately prescribing ABs [8]. POCTs could not only improve the use of antibiotics but also reduce patient pressure for antibiotic prescriptions [9]. In a study by Llor et al., general practitioners in Spain were strongly influenced by POCT results in the decision to prescribe antibiotics [10]. Gunnarsson et al., reported that in Australia, 40% of patients with uncomplicated acute sore throat who were prescribed antibiotics had bacterial AP before testing, while this proportion increased to 97% after testing [11]. No data have been published as of yet on the use of POCTs in primary care in Germany.

The objective of this present study was to evaluate the administration of POCTs to detect Group A streptococci infection among AP patients in primary care.

## 2. Methods

A study was conducted in 1257 primary care practices between February 2021 and March 2022 in Germany. Two questionnaires were administered to physicians. The questions contained in the first questionnaire are displayed in Table 1. This questionnaire contained six questions about the use of throat swab tests (TSTs) and asked for the physicians’ opinions about their importance in everyday practice for the correct diagnosis of Group A Streptococci (GAS), for justifying to patients whether an antibiotic is needed and for achieving a more sensible use of antibiotics and the prevention of bacterial resistance.

The second questionnaire was developed for the physicians to include the TST results of ten AP patients. For each tested patient, the physicians were required to indicate if the test was positive or negative and if antibiotic or symptomatic therapy was prescribed (Table 2).

## 3. Results

### 3.1. Results of the Physician Survey

Of the 1257 physicians, 750 (59.6%) used throat swab tests (TSTs). Of these, 24.7% used a TST before prescribing an antibiotic, 39.4% used TSTs in cases of severe sore throat, 39.9% used TSTs in cases of long-lasting pharyngitis and 24.5% used TSTs in other cases. Additionally, 41.9% responded that they frequently use TSTs in a defined patient group, for example, the elderly or children.

In total, 82.6% considered the adoption of TSTs in everyday practice to be important for the diagnosis of Streptococci Group A, 80.2% considered it important to justify to patients whether an antibiotic is needed and 90.2% considered it important or very important for achieving a more sensible use of antibiotics and the prevention of bacterial resistance.

### 3.2. Diagnosis of GAS and Antibiotic Prescriptions Based on Point-of-Care Testing

**Figure 1:**
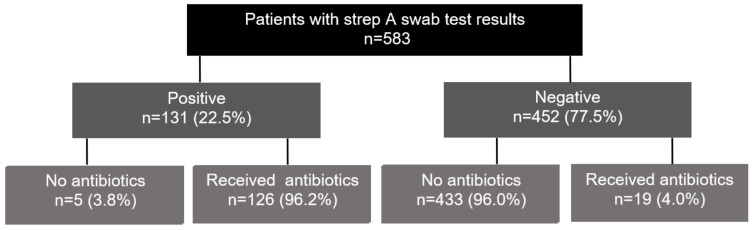
Proportion of patients with negative or positive POCT results and those who were prescribed antibiotics.

A total of 73 PCPs presented TST results which were available for 583 patients (8.0 patients per practice). Of these, 131 (22.5%) tested positive for strep throat, and 126 of 131 (96.2%) patients who tested positive received an AB prescription. Of the 452 patients who tested negatively, 19 (4.0%) received an AB prescription. In total, 127 (21.8%) of 582 study patients were treated with Abs (Figure 1).

**Figure 2:**
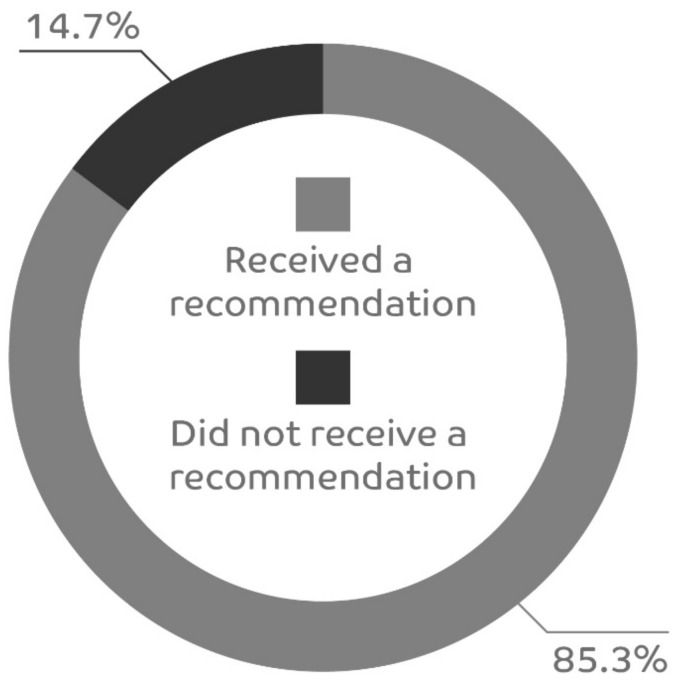
Proportion of patients who received a recommendation for symptomatic treatment.

Feedback was received for 464 patients for the question “Was symptomatic treatment recommended”, and 396 (85.3%) of patients were given a recommendation for symptomatic treatment (Figure 2). The majority of physicians (201/324, 62%) favored local symptomatic treatment with lozenges and sprays, and 11% (36/324) recommended a combination of symptomatic and systemic therapy.

## 4. Discussion

In this study conducted in 1257 PCPs, 80% of the physicians used TSTs, mainly in cases of severe sore throat or long-lasting pharyngitis. At least 80% of the physicians considered the use of TSTs to be important for the diagnosis of GAS, achieving a more sensible use of antibiotics, the prevention of bacterial resistance and for justifying to patients the decision to use or not to use antibiotics. Finally, 22.5% of AP patients tested positive for GAS, and since 21.8% received an AB prescription.

The positive opinion of the physicians about POCTs is not surprising. First, the results of POCTs are available within minutes. Second, POCTs have a high level of diagnostic accuracy for GAS in both adults and children. Based on 48 studies, Lean et al., demonstrated a sensitivity of 86% and a specificity of 96% in both adults and children [12]. Stewart et al., also showed high specificity (93%) and sensitivity (91%) for POCTs in adults with bacterial pharyngitis [13]. Orda et al., could show that POCTs provide sufficient accuracy to guide antibiotic prescription in children with sore throat upon first presentation. Of 101 children with sore throat, 25.7% tested positive for GAS; of 148 children without sore throat, only one tested positive (specificity 99%) [14].

The proportion of patients with a positive GAS test and with an antibiotic prescription following a POCT in our study is in line with the published research. Papastergiou et al. (2018) found that 25.5% of patients tested positive for GAS in pharmacies in Canada [15], which is very similar to our proportion of 25.7%. In a randomized, controlled trial published by Worrall et al., 26.7% received an AB prescription [16]. In a pediatric emergency department in the United Kingdom, 24% of children with AP in the first year and 28% in the second year after the introduction of GAS POCTs received AB prescriptions [17].

Previous studies showed that use of POCTs in PCP offices was associated with a significant reduction in AB prescriptions. Cohen et al., performed a systemic review including five trials with 2545 patients with sore throat in primary care settings. Based on these data, the authors concluded a 25% reduction in prescribed antibiotics via the use of POCTs versus management based on clinical scoring [18]. Mantzourani et al., investigated the consequences of removing the requirement for POCTs from pharmacies in Wales. The inappropriate antibiotic supply rate increased from 27% to 63%, showing that the use of POCTs may result in fewer inappropriate antibiotic prescriptions for sore throat symptoms [19].

In our study, we could not investigate the reduction in AB prescriptions through the use of POCTs. However, when two studies from PCP settings in Germany are compared, this reduction can be assumed. In the present study, 23.9% of adult AP patients received an AB prescription. In the total population of AP patients in the study by Kern and Kostev, this proportion was 46% [3]. This means a decrease from 46 to approximately 24% (−48%).

Van der Velden et al., described differences in respiratory tract infection diagnostic testing and the prescription of antibiotics between 18 European countries. Although ABs were prescribed more often than considered appropriate and POCTs were used rarely, PCPs had high confidence in their antibiotic-prescribing decisions [20].

A recent study (HALS) comparing the influence of a guideline recommending the use of different diagnostic scores (Centor Score, McIssac Score, and Fever–Pain Score) or in combination with an additional POCT showed the better adherence of physicians to negative POCTs and hence fewer prescriptions of antibiotics for sore throat [21].

The principle aim of the German guideline is to avoid the overuse of antibiotics for the symptoms of sore throat and thus reduce nonindicated antibiotics in patients with trivial respiratory tract infections. The recommended treatments are non-medicated lozenges or medicated lozenges containing anesthetics or non-steriodal anti-inflammatory drugs (NSAIDs) and systemic NSAIDs (DEGAM Leitlinie S3: Halsschmerzen) [22].

## 5. Study Limitations

Our study has some limitations which should be listed here. First, the analyses were only descriptive, and no statistical hypotheses were tested. Moreover, several variables were not available they were not collected through the questionnaire. These variables include the age and sex of patients and the drug, the daily dose and the duration of therapy of the prescribed antibiotics. Finally, results of our study may not be generalizable for all physicians, especially physicians in other countries.

## 6. Conclusions

Our study shows that the use of POCTs in patients with sore throat in PCP practices resulted in high levels of physician satisfaction with the outcome of less inappropriate AB use.

The use of pharyngeal swab tests in patients with acute pharyngitis in German primary care practices was considered important by physicians and could strongly reduce the nonindicated use of antibiotics. Furthermore, the use of POCTs enables patients and physicians to make joint decisions for symptom-focused treatment. Further data would be desirable to determine whether reimbursements for the tests could lead to more frequent use and fewer antibiotic prescriptions, thus enhancing antibiotic stewardship.

## Figures and Tables

**Table 1:** Questions included in the first questionnaire.

Question	Possible Answers
Do you use throat swab tests in your practice to detect a-streptococci?	yes, no
If yes: in which cases do you use throat swab tests for sore throats?	always before prescribing an antibiotic, in case of particularly severe sore throat, for particularly long-lasting sore throats, others
If yes: is there a patient group for whom you frequently use the throat swab tests (children, elderly, etc.)?	yes, no, no specification
How important do you consider the integration of a throat swab test in the everyday practice for the correct diagnosis of Streptococci Group A?	very important, important, neither, rather unimportant, unimportant
How important do you consider the performance of a throat swab test to justify to patients whether or not an antibiotic is needed?	very important, important, neither, rather unimportant, unimportant
How important do you consider throat swab testing for more sensible use of antibiotics and the prevention of bacterial resistance?	very important, important, neither, rather unimportant, unimportant

**Table 2:** The structure of the second questionnaire.

TST number 1-583	Result		Antibiotic prescription		Recommendation of symptomatic therapy		If yes, which?	Comment
	positive	negative	yes	no	yes	no		

## Data Availability

The data and the code used for this study are available from the corresponding author upon reasonable request.
